# Duality of the SVIL expression in bladder cancer and its correlation with immune infiltration

**DOI:** 10.1038/s41598-023-41759-1

**Published:** 2023-09-05

**Authors:** Zhenyu Nie, Na Guo, Yanling Peng, Yuanhui Gao, Hui Cao, Shufang Zhang

**Affiliations:** Central Laboratory, Affiliated Haikou Hospital of Xiangya Medical College, Central South University, Haikou, 570208 Hainan China

**Keywords:** Bladder cancer, Tumour immunology

## Abstract

SVIL is a member of the villin/gelsolin superfamily and is responsible for encoding supervillin. It has been reported to be closely related to the occurrence and development of various tumors. However, the mechanism of SVIL in bladder cancer has not been reported yet. In this research, we evaluated the relationship between SVIL expression and bladder cancer in public dataset and examined the expression of SVIL in bladder cancer cell lines, tissue microarrays and patients in our cohort. Our work determined that the expression of SVIL in bladder cancer tissue was significantly lower than that in normal tissue. However, in bladder cancer tissues, the high expression of SVIL is significantly associated with poor prognosis. This kind of duality is very novel and has great research value. The expression level of SVIL can well predict the survival time of bladder cancer patients, and is an independent risk factor of bladder cancer patients. The expression of SVIL is also closely related to the immune tumor microenvironment of bladder cancer. Our research provides a basis for personalized therapeutic targets for bladder cancer.

## Introduction

Bladder cancer is one of the most common malignancies of the urinary system. According to cancer statistics released in 2022, 81,800 new cases and 17,100 deaths from bladder cancer were predicted in the United States^1^, which is a slight decline from 2021^2^. There were 82,300 new cases of bladder cancer and 33,700 deaths in China in 2015^3^. Bladder cancer is a broad concept that includes low-risk non-muscle invasive bladder cancer (NMIBC) and high-risk muscle-invasive bladder cancer (MIBC). The most problematic for low—and intermediate-risk NMIBC patients is the high recurrence rate, with a five-year recurrence free survival rates of only 43% and 33%, respectively^4^. Metastasis of bladder cancer is a disaster that 50–70% of MIBC patients faced, and the extremely high rate of metastasis makes a 5-year overall survival (OS) of only 4.8%^5^. Numerous studies have shown that a variety of gene mutations, abnormal gene expression, and/or activation states of signaling pathways are essential for tumor development^6^. Therefore, more in-depth and detailed studies on the molecular mechanisms of bladder cancer pathogenesis are urgently needed to identify biomarkers for early screening and identify potential therapeutic targets.

SVIL is a member of the villin/gelsolin superfamily and is responsible for encoding supervillin, a 205 kDa F-actin-binding protein^7,8^. SVIL has been implicated in several aspects of the initiation and progression of various tumors, including cell survival, migration, and invasion^9,10^. For example, SVIL can promote cancer cell survival by regulating the level of p53^11^, or in response to signals of intratumoral hypoxia, thereby increasing its own expression, ultimately leading to tumor metastasis and poor survival in liver cancer^10^. In addition, SVIL can promote tumor development in hepatocellular carcinoma by promoting angiogenesis, which can be inhibited by targeting SVIL, and is considered as a potential therapeutic biomarker^12^. However, the expression of SVIL in bladder cancer and its functional relationship with tumors has not yet been reported.

In this study, we used multiple public databases, bladder cancer cell lines, tissue microarrays, and clinical specimens from our cohort to comprehensively analyze the correlation between SVIL expression and clinicopathological features, determine the diagnostic and prognostic value of SVIL in bladder cancer. In addition, we investigated the possible pathogenic mechanisms of SVIL by performing gene set enrichment analysis (GSEA) on the SVIL high and low expression groups. Finally, since immune cells in the tumor microenvironment (TME) are important factors that interfere with the biological processes of tumorigenesis, development, and survival, we also discussed the relevance of SVIL to the infiltration of immune cells in bladder cancer. In general, this study not only highlights the importance of SVIL in bladder cancer but also demonstrates its potential as a prognostic biomarker and therapeutic target for bladder cancer.

## Methods and materials

The studies involving human participants were reviewed and approved by the ethics committee of Haikou Hospital affiliated to Xiangya Medical College of Central South University. The patients/participants provided their written informed consent to participate in this study. The human bladder cancer cell lines and human immortalized urothelial cell line used in this study are commercial products with ethical exemption rights. All of the cell lines were purchased from the China Centre for Type Culture Collection (Shanghai, China). In addition, all methods in this article were performed in accordance with the relevant guidelines and regulations.

### TIMER database analysis

Tumor Immune Estimation Resource (TIMER, http://timer.cistrome.org) is an online database used for the comprehensive analysis of tumor-infiltrating immune cells and different gene expression levels in different cancer types^13^. We used the TI MER database to study the differential expression of SVIL between tumor and normal tissues across various cancer types.

### RNA-sequencing data and bioinformatic analysis

Normalized RNA-seq data and corresponding clinicopathological information of 412 bladder cancer (BLCA) tumor tissues and 19 surrounding normal tissues were acquired from The Cancer Genome Atlas database (https://portal.gdc.cancer.gov/) (TCGA-BLCA dataset). The data format of level 3 HTSeq transcripts per million (TPM) were downloaded. The main clinicopathological features of the bladder cancer patients are shown in Table 1. Some patients had incomplete clinical information and were only excluded when comparing the specific clinical factors that they lacked. In addition, to validate the SVIL mRNA expression in patients with bladder cancer, the raw gene profile of GSE13507^14,15^ was downloaded from the Gene Expression Omnibus (GEO) database (https://www.ncbi.nlm.nih.gov/geo/). Clinical information of GSE13507 dataset in GEO database is shown in Table 2.

**Table 1:** Clinical information of TCGA-BLCA dataset in TCGA database.

Characteristics	Patients (N = 412)	
	n	%
Age category		
< 60 y	88	21.36%
≥ 60 y	324	78.64%
Gender		
Male	304	73.79%
Female	108	26.21%
Pathologic stage		
Stage I	2	0.49%
Stage II	131	31.80%
Stage III	141	34.22%
Stage IV	136	33.01%
Not report	2	0.49%
T stage		
Ta	1	0.24%
T1	3	0.73%
T2	120	29.13%
T3	196	47.57%
T4	59	14.32%
Tx	33	8.01%
N stage		
N0	239	58.01%
N1	47	11.41%
N2	76	18.45%
N3	8	1.94%
Nx	42	10.19%
M stage		
M0	196	47.57%
M1	11	2.67%
Mx	205	49.76%
Overall survival		
Alive	229	55.58%
Dead	182	44.17%
Not report	1	0.24%
Smoker		
Yes	222	53.88%
No	190	46.12%

**Table 2:** Clinical information of GSE13507 dataset in GEO database.

Characteristics	Patients (N = 165)	
	n	%
Age category		
< 60 y	46	27.88%
≥ 60 y	119	72.12%
Gender		
Male	135	81.82%
Female	30	18.18%
Grade		
Low	105	63.64%
High	60	36.36%
T stage		
Ta	24	14.55%
T1	80	48.48%
T2	31	18.79%
T3	19	11.52%
T4	11	6.67%
N stage		
N0	150	90.91%
N1	8	4.84%
N2	5	3.03%
M stage		
M0	158	95.76%
M1	7	4.24%
Overall survival		
Alive	96	58.18%
Dead	69	41.82%

### Cell culture

Bladder cancer cell lines (T24, RT4, 5637, UMUC-3, and J82) and human ureteral epithelial immortalized cells (SV-HUC-1) were purchased from the China Centre for Type Culture Collection (Shanghai, China), and all of the cell lines have the STR certificate. All cells were cultured in an incubator containing 5% CO_2_ and 95% air at 37 ℃. All cell culture media and FBS (30,044,333) were obtained from Gibco (Grand Island, NY, USA). J82 and UMUC-3 cells were cultured in DMEM (10% FBS) (10,569,010), T24 and 5637 cells were cultured in RPMI 1640 (10% FBS) (11,879,020), RT4 cells were cultured in McCoy’s 5A (16,600,082) (10% FBS) medium, and SV-HUC-1 cells were cultured in F-12 K (21,127,022) (10% FBS) medium.

### Patients and clinical tissue samples

8 pairs of tumor tissues and corresponding surrounding normal tissues of patients with primary bladder cancer were collected from Haikou Hospital (Haikou People's Hospital, Haikou, China) affiliated to Xiangya Medical College of Central South University from 2020 to 2022. Surrounding normal tissues with a margin > 3 cm were extracted from the tumor and evaluated microscopically to exclude dysplastic cells. None of the patients with bladder cancer had other malignant tumors and underwent biopsy. The pathological diagnosis was bladder cancer. None of the patients had received neoadjuvant radiotherapy, chemotherapy, or other special treatments before surgery. All patients signed an informed consent form, and this study was reviewed and approved by the Medical Ethics Committee of the Haikou People's Hospital (ethics file number: ZY-IRB-FOM-037).

### Quantitative real-time polymerase chain reaction

Total RNA was extracted from tissues using RNA isolater Total RNA Extraction Reagent (Vazyme, Nanjing, China, R401-01). Reverse transcription to cDNA was performed using HiScript^®^III All-in-one RT SuperMix Perfect for qPCR (Vazyme, R333). cDNA was amplified with an Applied Biosystems QuantStudio 5 Real-Time PCR instrument (Thermo Fisher Scientific, Waltham, MA, USA) using ChamQ Universal SYBR qPCR Master Mix (Vazyme, Q711). The primer sequences used were as follows:

SVIL forward prime:5’-GACACCCCTCGATACATGAGA-3’.

SVIL reverse prime:5’-CGGAGGTTTCTGTGCAGTATT-3’.

β-actin forward prime:5’-CATGTACGTTGCTATCCAGGC-3’.

β-actin reverse prime:5’-CTCCTTAATGTCACGCACGAT-3’.

The 2^−∆∆Ct^ comparative method was applied to calculate the relative SVIL mRNA expression.

### Tissue microarray and immunohistochemistry (IHC) staining

The tissue microarray of bladder cancer and normal tissues surrounding the tumor were purchased from Bioaitech Biotechnology Co., Ltd. (Xi’an, China, U812101). The microarray contained 70 cases of urothelial carcinoma and 11 cases of paraneoplastic tissue, of which 7 cases were paraneoplastic and tumor-matched (Table 3).

**Table 3:** Clinical information of tissue microarray.

Characteristic		Total number (N = 70)	
		Numbers (n)	%
Ages	< 60 y	30	42.86%
	≥ 60 y	40	57.14%
Gender	Male	60	85.71%
	Female	10	14.29%
Tumor size (shortest length)	≤ 3.0 cm	35	50.00%
	> 3.0 cm	15	21.43%
	Unable to calculate/not recorded	20	28.57%
T-stage	Ta	23	32.86%
	T1	27	38.57%
	T2	12	17.14%
	T3	8	11.43%
N-stage	N0	70	100%
M-stage	M0	70	100%
Clinical stage	0	23	32.86%
	I	27	38.57%
	II	12	17.14%
	III	8	11.43%

IHC staining of the tissue microarray was performed in the following steps: dewaxing, blocking endogenous peroxidase, serum blocking, primary antibody binding, secondary antibody binding, DAB coloration, restaining the nucleus, and dehydration seal. The results were obtained using the H-score system, which is a scoring method widely used in immunohistochemical pathology research and can accurately evaluate the positive rate of each region. SVIL polyclonal antibody was purchased form Invitrogen (California, USA, PA5-5145).

### Enrichment analysis of GSEA

Gene set enrichment analysis (GSEA) was used in the present study, which can evaluate whether a previously defined set of genes has statistically significant and consistent differences between two biological states^16^. The tumor samples were divided into low and high SVIL groups according to the data downloaded from TCGA database. Pathways enrichment was analyzed based on the adjusted P-value and normalized enrichment score (NES).

### Immune infiltration analysis

Gene markers for 24 immune cells were derived from a previous study^17^. The level of tumor immune infiltration was identified using the single-sample GSEA (ssGSEA) method with the GSVA R package based on TCGA-BLCA data sets. Correlation analysis between SVIL and these 24 immune cell types was performed using the Spearman correlation test. Graphs and figures were generated using ggplot2 R package.

### Statistically analysis

Bioinformatics analysis was performed using R version 3.6.3. The difference expression in SVIL expression between normal and tumor tissues was analyzed using the Wilcoxon signed-rank test and one-way ANOVA. The Fisher’s exact test, chi-square test, Wilcoxon signed-rank test, and logistic regression were used to estimate the correlation between SVIL expression and clinicopathological features. Receiver operating characteristic (ROC) curve analysis was applied, with the area under the curve (AUC) used as an index of diagnostic accuracy. In addition, we used the Kaplan–Meier method and Cox regression to evaluate the role of SVIL expression in prognosis. In Cox regression analysis, statistically significant variables in univariate Cox regression were furtherly enrolled into multivariate Cox regression. Statistically significance was set at p < 0.05.

### Ethics statement

The studies involving human participants were reviewed and approved by the ethics committee of Haikou Hospital affiliated to Xiangya Medical College of Central South University. The patients/participants provided their written informed consent to participate in this study.

## Result

### Transcriptional level of SVIL in patients with bladder cancer

**Figure 1:**
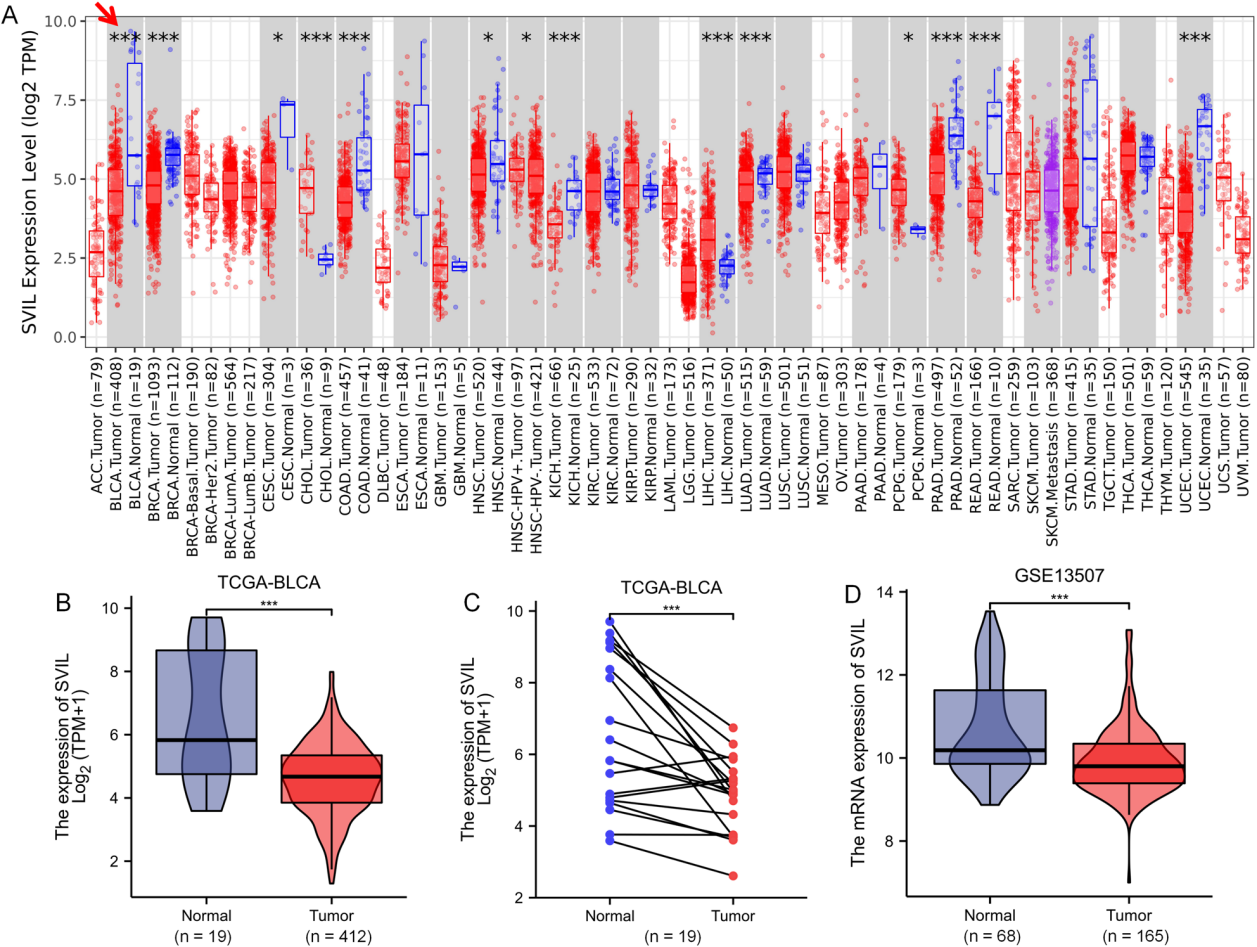
Transcriptional level of SVIL in TCGA-BLCA and GSE13507. (**A**) The expression level of SVIL in pan-cancer, red arrow points the bladder cancer tissues out; (**B**) The expression level of SVIL in TCGA-BLCA; (**C**) Expression of SVIL in paired bladder cancer tissues (TCGA-BLCA); (**D**) The expression level of SVIL in GSE13507. *P < 0.05; **P < 0.01; ***P < 0.001.

To determine the overall expression levels of SVIL in different malignancies, we first analyzed SVIL expression in different cancer types using the TIMER database. The results showed that SVIL exhibited different expression profiles in different types of tumor tissues. The mRNA expression of SVIL in bladder cancer was significantly downregulated compared with that in normal tissues (red arrow) (Fig. 1A).

To further clarify the differences in SVIL expression between bladder cancer and normal tissues, we collected RNA-seq data and clinical information from 19 paraneoplastic and 412 bladder cancer tissues from the TCGA-BLCA dataset. The results showed that SVIL was significantly downregulated in BLCA tissues (p < 0.001, Fig. 1B), and in addition, we analyzed the SVIL expression levels in 19 groups of bladder cancers and their matched adjacent normal tissues, which showed lower expression in BLCA tissues compared to normal tissues (p < 0.001, Fig. 1C). Furthermore, to validate the above results, we downloaded microarray data from the GEO database, namely GSE13507, which showed that SVIL was significantly downregulated in BLCA tissues compared to normal tissues (p < 0.001, Fig. 1D).

### Validation in bladder cancer cell lines, clinical specimens and tissue microarray

**Figure 2:**
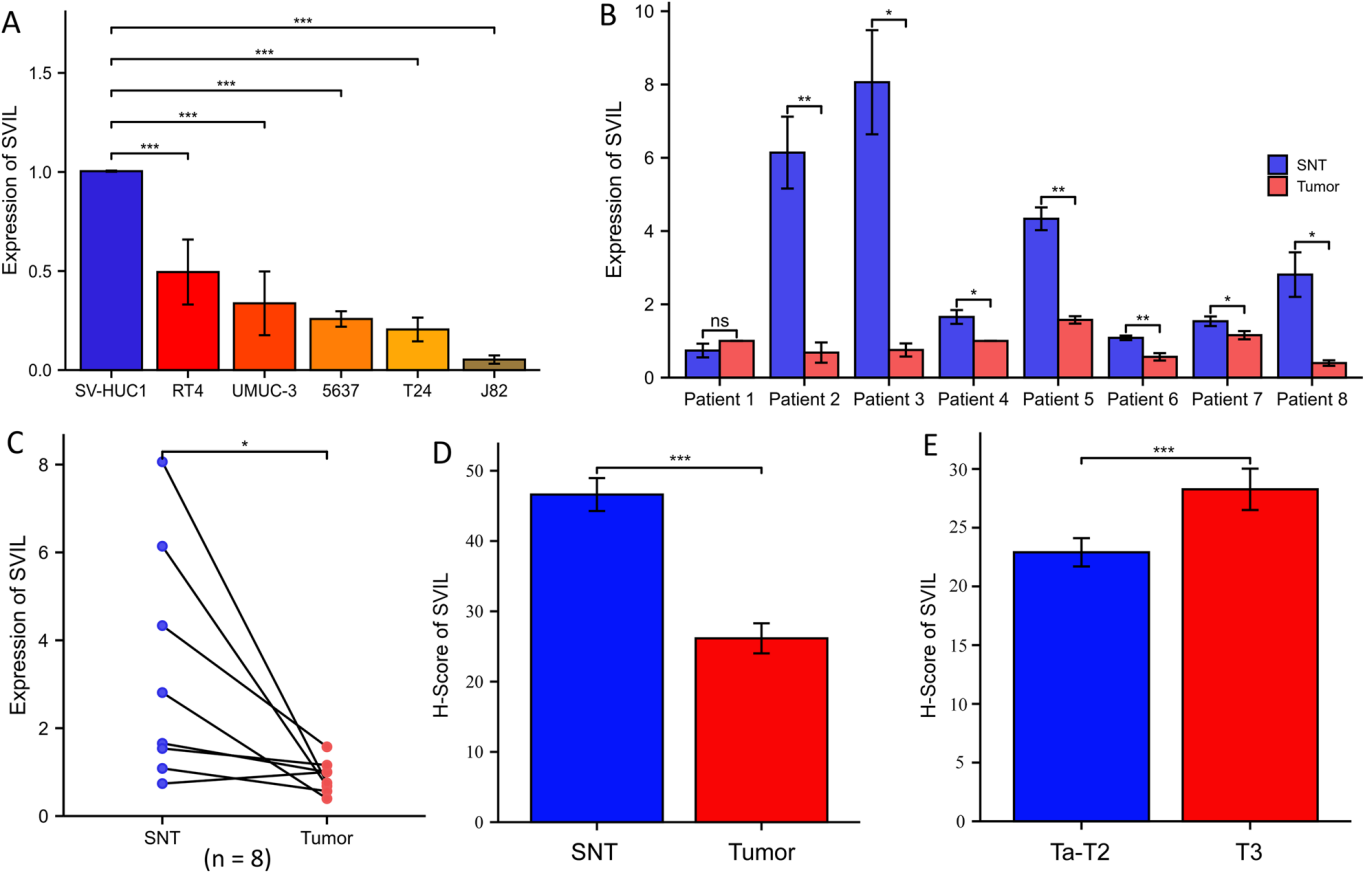
The expression of SVIL in bladder cancer cell lines and clinical patients tissues. (**A**) The expression of SVIL in bladder cancer cell lines, the expression of SVIL in each cell line are compared with its β-actin, and normalized to SV-HUC-1. (**B**) and (**C**) The expression of SVIL in 8 pairs of bladder cancer patients tissues, similarly, the expression of SVIL in each cell line are compared with its β-actin. (**D**) H-Score of SVIL expression between SNT and tumor in tissue microarray. (**E**) H-Score of SVIL expression between Ta-T2 and T3 in tissue microarray. The 2^−∆∆Ct^ comparative method was applied to calculate the relative SVIL mRNA expression. *P < 0.05; **P < 0.01; ***P < 0.001. *SNT* surrounding normal tissue.

We verified the expression of SVIL in bladder cancer cell lines (T24, RT4, 5637, UMUC-3, and J82) and human ureteral epithelial immortalized cells (SV-HUC-1). The results showed that SVIL expression in SV-HUC-1 cells was significantly higher than that in all bladder cancer cell lines (Fig. 2A). We verified the expression of SVIL in bladder cancer tissue and its corresponding surrounding normal tissue (SNT) (n = 8) (Fig. 2B,C). Similarly, the results showed that the expression of SVIL in SNT was significantly higher than that in bladder cancer.

Similarly, we validated the expression of SVIL in tissue microarrays and compared the differences between groups through H-Score. Results shown in Fig. 2D,E, the expression of SVIL in surrounding normal tissue is higher than that in bladder cancer tissues. But interestingly, SVIL expression in bladder cancer tissues of higher T-stage group is higher than that of lower T-stage group.

### In bladder cancer, upregulated SVIL correlates with poor clinicopathological features of bladder cancer

**Figure 3:**
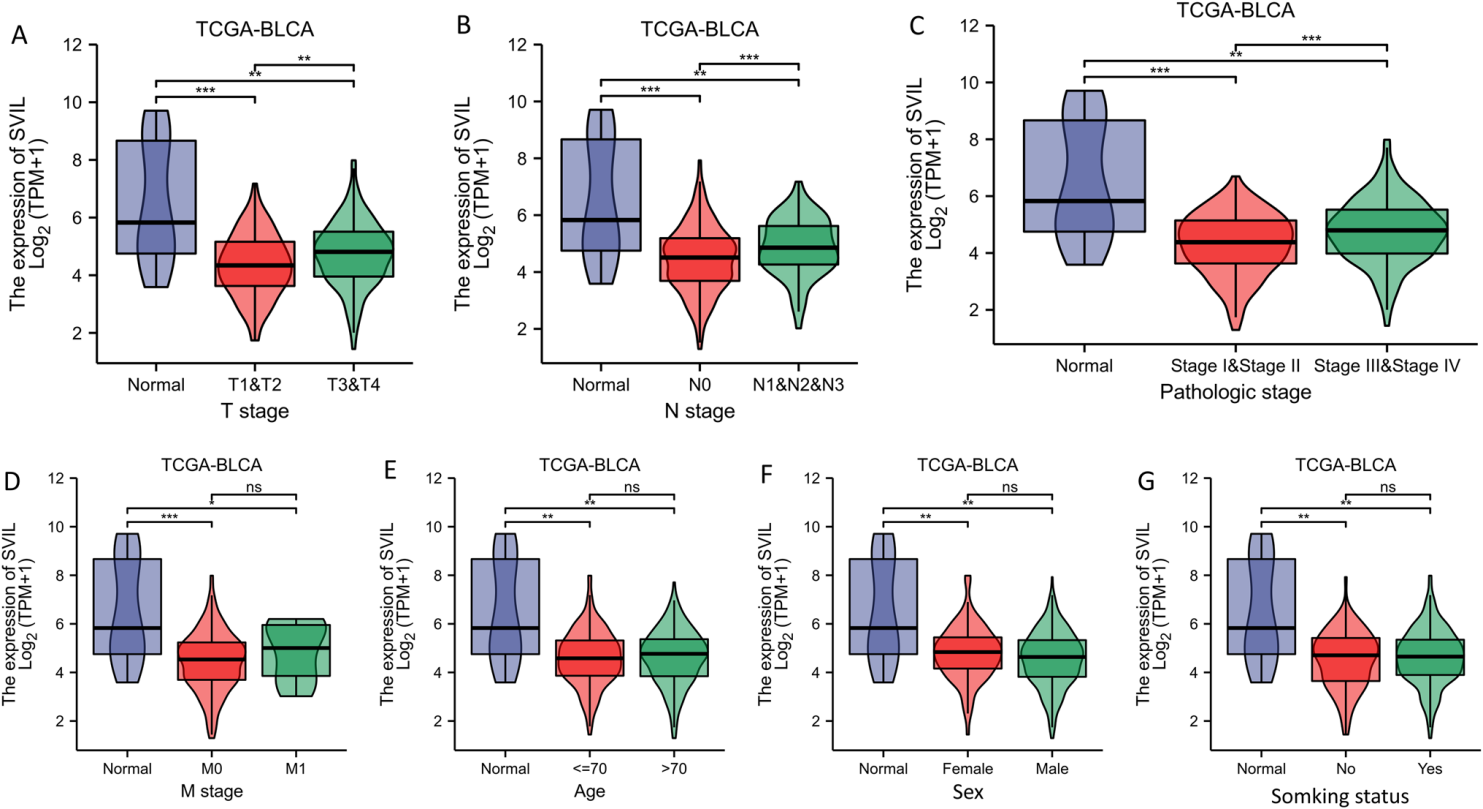
The expression of SVIL correlates with clinicopathological features of bladder cancer. The expression of SVIL correlates with T stage (**A**), N stage (**B**), and pathologic stage (**C**). These clinicopathological characteristics were negatively correlated with the expression of SVIL in bladder cancer patients. The expression of SVIL is not related to M stage (**D**), age (**E**), sex (**F**), and smoking status (**G**) in bladder cancer patients. *P < 0.05; **P < 0.01; ***P < 0.001; *ns* no significance.

Inspired by the results in Fig. 2E, we decided to compare the relationship between SVIL and various clinical features of bladder cancer. We collected clinical information and gene expression data from TCGA database for all patients and divided the patients into high and low expression groups based on the average expression value relative to SVIL. By assessing the association between SVIL expression and different clinicopathological characteristics of bladder cancer patients, the results showed that although SVIL mRNA was significantly lower in all bladder cancer tissues than in normal tissues, elevated SVIL mRNA expression in the tumor tissues of bladder cancer patients was associated with higher T-stage (p < 0.01), N-stage (< 0.01), and pathological stage (p < 0.001) (Fig. 3A–C). There was no significant correlation (p > 0.05) between M stage, age, smoking status, and sex (Fig. 3D–G). Accordingly, we tentatively concluded that the mRNA expression level of SVIL showed a duality in bladder cancer; that is, the expression in normal tissues was significantly higher than that in all categories of bladder cancer tissues, but in high-grade bladder cancer, the expression of SVIL was higher than that in low-grade bladder cancer tissues.

### SVIL expression can be a potential biomarker for bladder cancer patients

**Figure 4:**
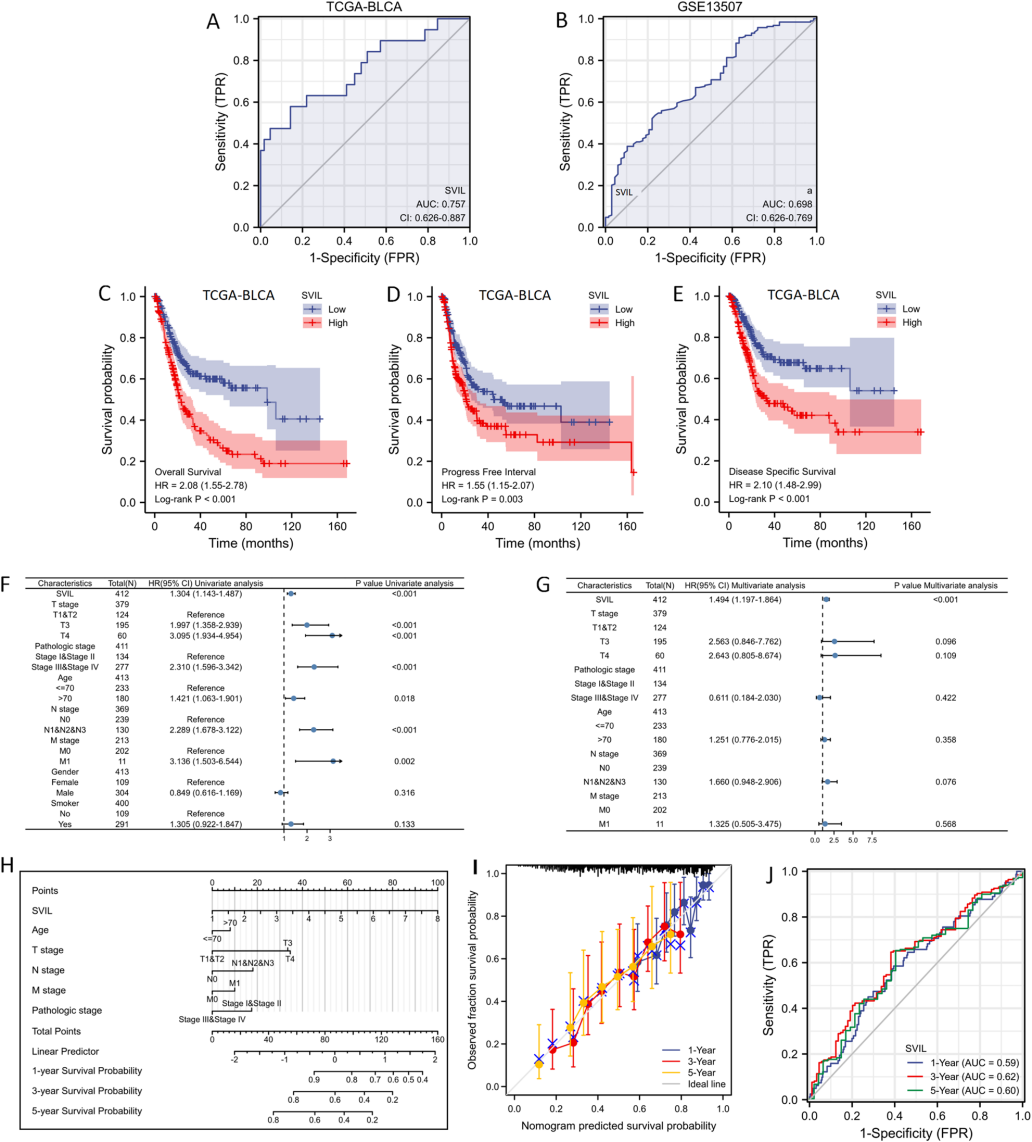
SVIL expression can predict prognosis bladder cancer patients. ROC analysis of SVIL mRNA expression level in TCGA-BLCA (**A**) and GSE13507 (**B**). Kaplan–Meier survival analysis based on the TCGA-BLCA reveals the OS (**C**), PFI (**D**), and DSS (**E**) with SVIL expression in TCGA-BLCA. Univariate (**F**) and multivariate (**G**) Cox regression analyses showed that elevated SVIL expression was an independent risk factor for prognosis in TCGA-BLCA. Nomogram (**H**), calibration curve (**I**), and ROC curve (**J**) were plotted to evaluate SVIL to predict survival time in TCGA-BLCA. *AUC* area under the curve, *ROC* receiver operating characteristic.

ROC analysis was used to assess the validity of SVIL mRNA expression levels in differentiating bladder cancer from normal tissues with an estimated AUC of 0.757 (95% CI 0.626–0.887) in the TCGA-BLCA dataset (Fig. 4A), and in the GSE13507 dataset to assess the expression of SVIL to differentiate bladder cancer tissues from normal tissues in the GSE13507 dataset, the AUC of SVIL was 0.698 (95% CI 0.626–0.769, Fig. 4B).

To further validate the correlation between SVIL expression and prognosis, we performed Kaplan–Meier survival analysis based on the TCGA-BLCA dataset, as shown in Fig. 4C–E. Among patients with diagnosed bladder cancer, high SVIL expression was significantly and positively associated with poorer OS (HR = 2.08, 95% CI 1.55–2.78, p < 0.001, Fig. 4C). Similarly, we also observed that high SVIL expression was significantly associated with poorer progression-free interval (PFI) (HR = 1.55, 95% CI 1.15–2.07, p = 0.003, Fig. 4D) and disease-specific survival (DSS) (HR = 2.10, 95% CI 1.48–2.99, p < 0.001, Fig. 4E). Univariate and multivariate Cox regression analyses showed that elevated SVIL expression was an independent risk factor for prognosis in bladder cancer (Fig. 4F,G). A nomogram was plotted to evaluate SVIL to predict survival time in bladder cancer patients (Fig. 4H), and the feasibility of this prediction method was verified by a calibration curve (Fig. 4I), and the ROC curve showed that the areas under the depressed line were 0.59, 0.62 and 0.60 at 1, 3 and 5 years, respectively (Fig. 4J).

### Predictive biological functions and pathways of SVIL in bladder cancer

**Figure 5:**
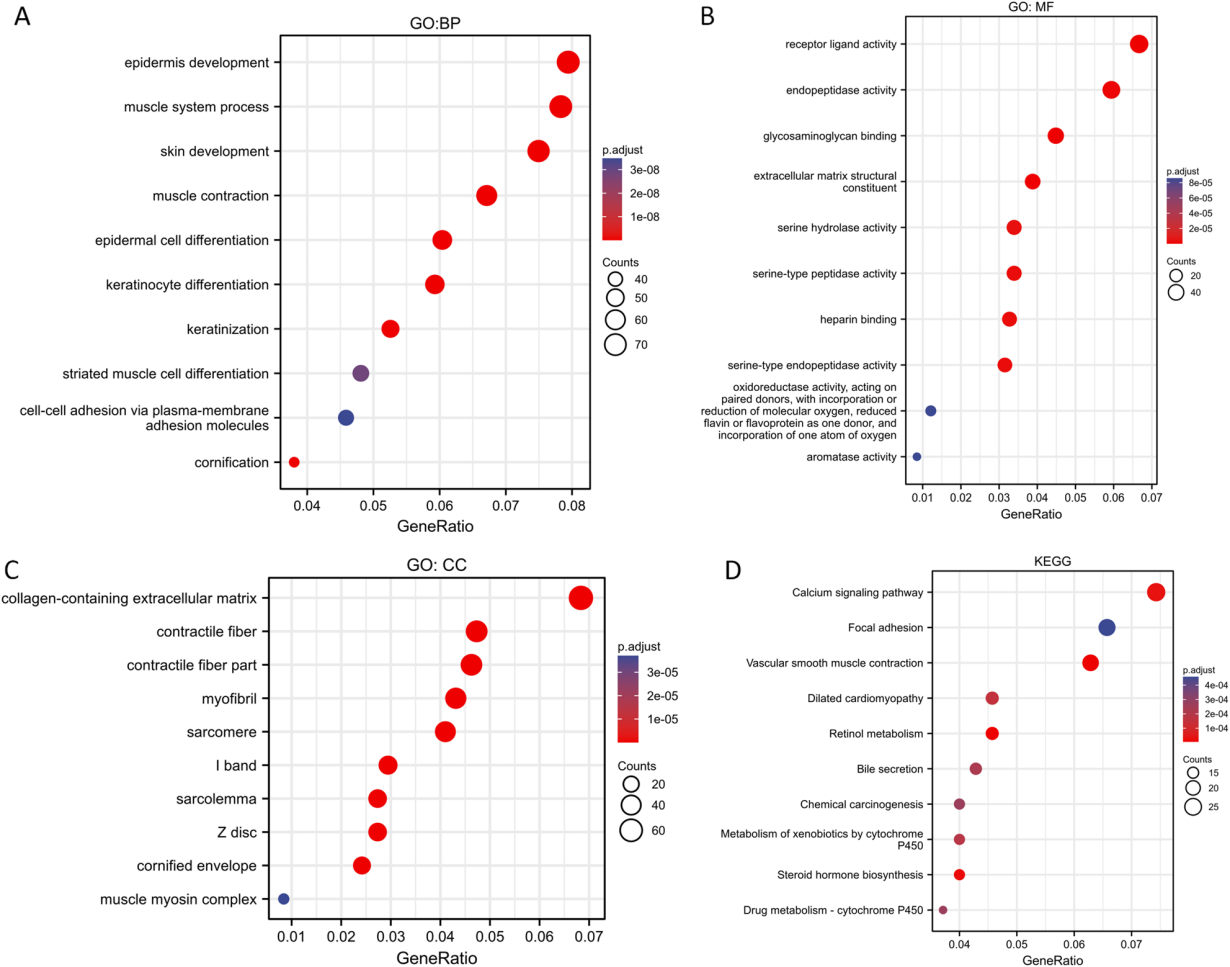
Enrichment analysis of SVIL in bladder cancer. (**A**–**C**) GO enrichment analysis of SVIL in TCGA-BLCA; (**D**) KEGG enrichment analysis of SVIL in TCGA-BLCA. *BP* biological process, *CC* cellular component, *GO* gene ontology, *KEGG* Kyoto encyclopedia of genes and genomes, *MF* molecular function.

Next, we analyzed potential biological functions and selected genes co-expressed with SVIL (|log_2_FC|> 1.0, p.adj < 0.05) for gene enrichment analysis. Analysis of GO terms for biological processes (BP) showed that epidermal development, muscle system process, skin development, strated muscle cell differentiation and cell–cell adhesion via plasma membrane adhesion molecules were significantly enriched (Fig. 5A). Analysis of molecular functions (MF) showed that receptor ligand activity, endopeptidase activity extracellular matrix structural constituents, and oxidoreductase activity were significantly enriched (Fig. 5B). Analysis of cellular constituents (CC) showed significant enrichment of collagen-containing extracellular matrix, contractile fibers, and sarcomeres (Fig. 5C). Kyoto Encyclopedia of Genes and Genomes (KEGG), is a database that integrates genomic, chemical, and system functional information (www.kegg.jp/kegg/kegg1.html)^18,19^. In this study, KEGG analysis showed that the calcium signaling pathway was the most significantly enriched pathway, and the rest of the enriched pathways were vascular smooth muscle contraction, chemical carcinogenesis, and metabolism of xenobiotics by the cytochrome P450 pathway (Fig. 5D). Overall, these results suggest that SVIL and its co-expressed genes may be collectively involved in myocyte and epithelial development and in pathways such as calcium signaling and chemical carcinogenesis, thereby regulating bladder carcinogenesis and progression.

**Figure 6:**
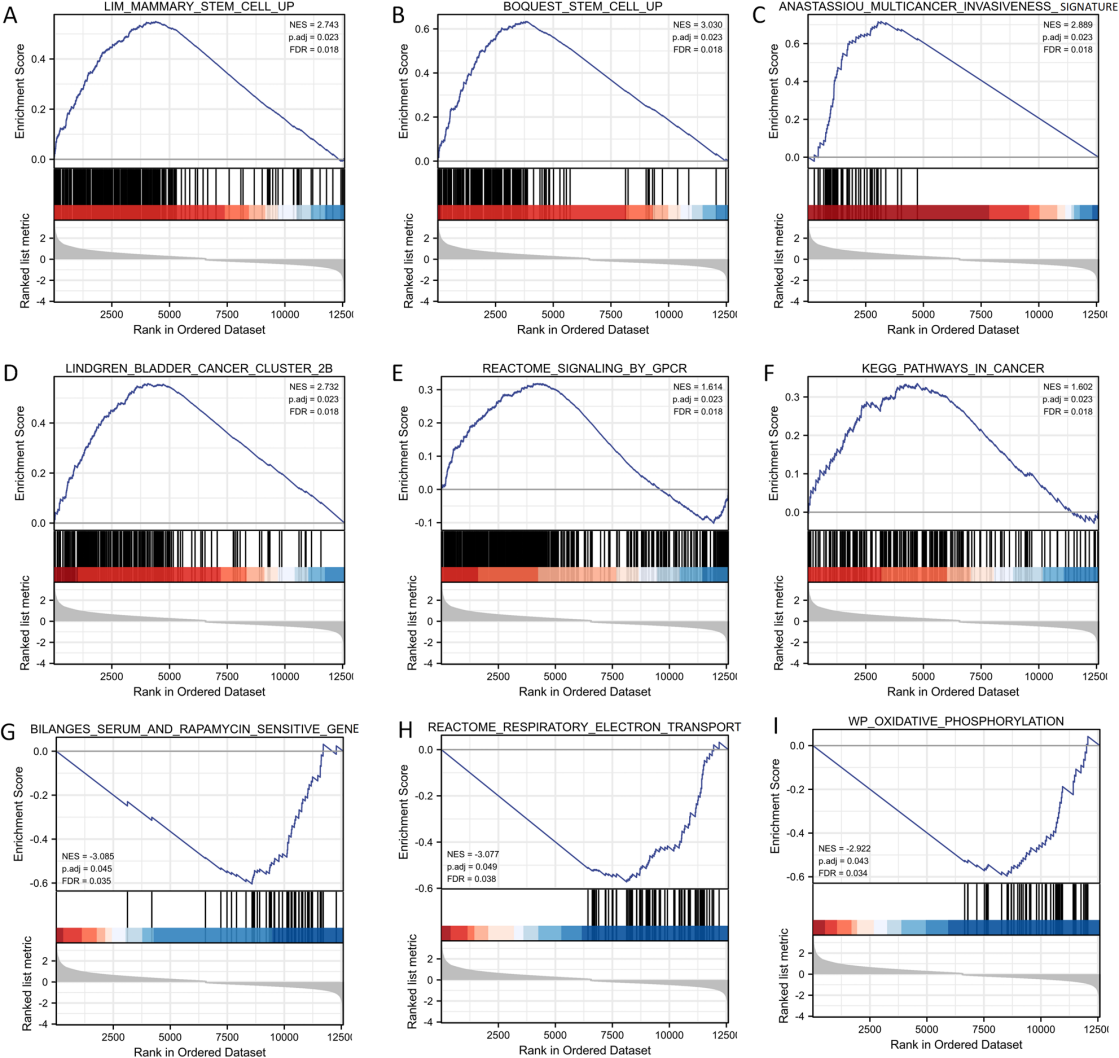
GSEA of SVIL in bladder cancer. (**A**–**I**) GSEA of SVIL in TCGA. *GSEA* gene set enrichment analysis.

In addition, GSEA analysis based on the normalized enrichment score (NES) and false discovery rate (FDR) q-value indicated the biological pathways that may be involved in the regulation between the SVIL high and low expression groups. As shown in Fig. 6 and, several signaling pathways were significantly enriched in the SVIL high expression group, including stem cell, multicancer invasiveness signature, bladder cancer cluster 2b, signaling by GPCR, and cancer pathways. In addition, serum and rapamycin-sensitive genes, respiratory electron transport, and oxidative phosphorylation were significantly enriched in the low SVIL expression group.

### Correlation of SVIL expression with immune cells and immune checkpoints in bladder cancer

**Figure 7:**
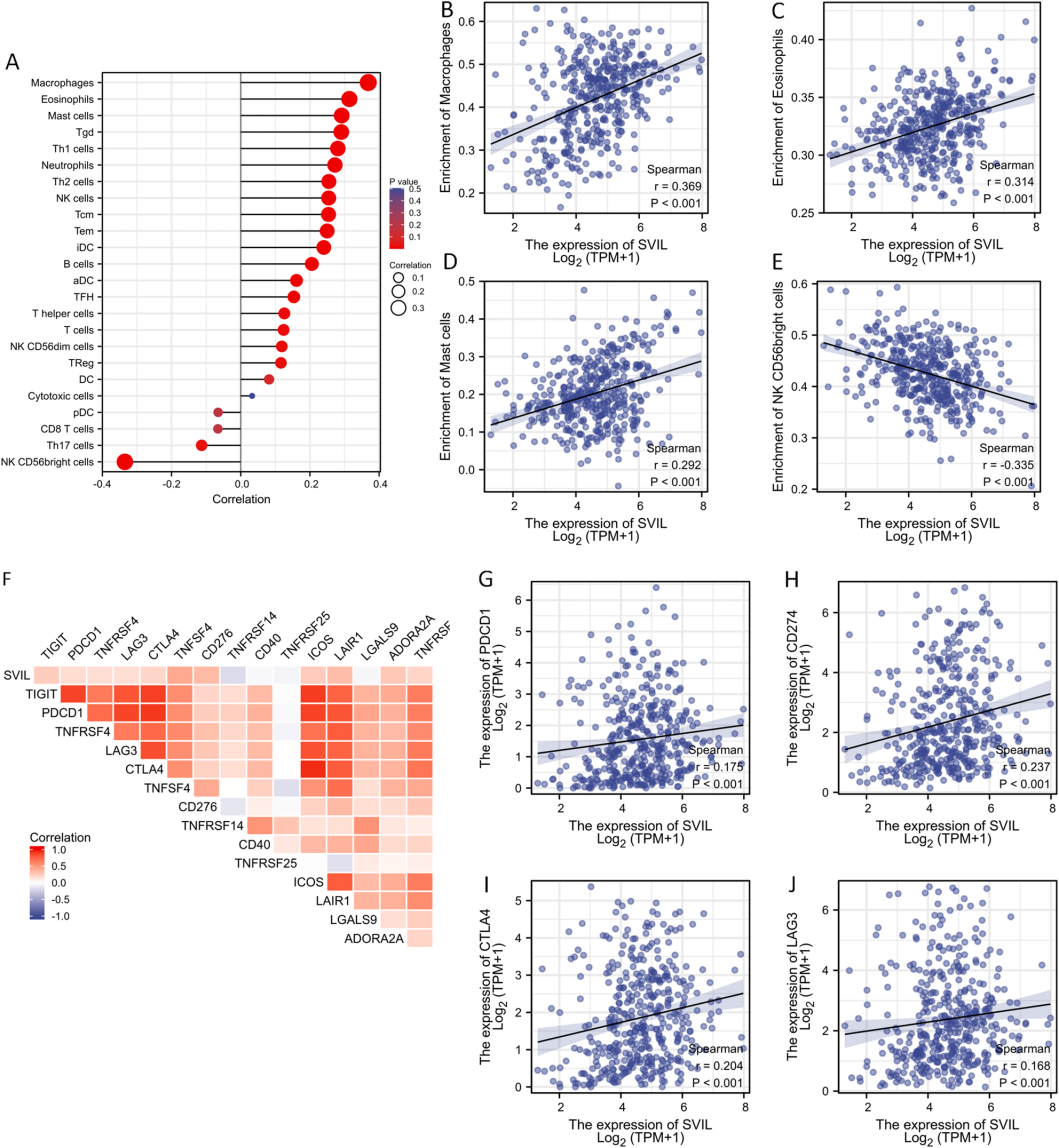
Correlation of SVIL expression with immune cells and immune checkpoints in bladder cancer. (**A**) The correlation between SVIL and 24 immune cell subpopulations in TCGA; Macrophage (**B**), eosinophils (**C**), mast cells (**D**), and CD56^bright^ cells (**E**) correlated with SVIL expression; F. The correlation of SVIL with immune checkpoint-related molecules; PDCD1 (**G**), CD274 (**H**), CTLA4 (**I**), and LAG3 (**J**) correlated with SVIL expression.

It has been reported that various immune cells infiltrating the tumor microenvironment significantly influence the prognosis of various tumors^17,20^. Therefore, we investigated the association between immune cell infiltration. We analyzed the correlation between SVIL and 24 immune cell subpopulations in bladder cancer using Spearman’s correlation (Fig. 7A) and found that SVIL was most significantly positively correlated with the level of macrophage infiltration (R = 0.369, p < 0.001) (Fig. 7B), followed by a significant correlation with eosinophils (R = 0.314, p < 0.001) (Fig. 7C) and mast cells (R = 0.292, p < 0.001) (Fig. 7D). In addition, SVIL expression was significantly negatively correlated with NK CD56bright cells (R = -0.335, p < 0.001) (Fig. 7E).

Subsequently, we analyzed the correlation of SVIL with immune checkpoint-related molecules (Fig. 7F), and we selected PD-1 and PD-L1-related molecules (PDCD1, CD274, CTLA4, and LAG3) (Fig. 7G,J) for correlation analysis with SVIL, and the results showed that SVIL was associated with PDCD1 (R = 0.175, p < 0.001), CD274 (R = 0.237, p < 0.001), CLTA4 (R = 0.204, p < 0.001), and LAG3 (R = 0.0.168, p < 0.001) were positively correlated.

## Discussion

Because of the insidious early symptoms of bladder cancer, the diagnosis of bladder cancer in clinical practice still relies mainly on cystoscopy, which is an invasive test and is thus resisted by most patients. However, there is still a lack of bladder cancer markers with sensitivity and specificity that meet the expectations. It also highlights the positive implications of identifying better biomarkers for early bladder cancer screening and for patients with recurrence. Since inactivation of tumor suppressor genes and activation of oncogenes are important causes of bladder cancer development^21^, exploration of the mechanisms of bladder cancer development to identify therapeutic targets for bladder cancer is also the focus of current research in the field of bladder cancer.

Although there are still relatively few studies on SVIL, it is clear from the available literature that SVIL is closely associated with the development and progression of a variety of tumors. In addition to being reported to be associated with hepatocellular carcinoma^10^, lung cancer^22^, and prostate cancer^23^, it is also thought to be closely associated with squamous cell carcinoma of the skin^24^. The human SVIL gene is localized to a single chromosomal locus at 10p11.2, a region that is deleted in some prostate cancers, implying that deletion of this gene may be closely associated with the development of some prostate cancers^25^. In addition, human SVIL mRNA is approximately 7.5 kb and is abundantly expressed in the HeLa S3 cervical cancer cell line, SW480 adenocarcinoma, and A549 lung cancer cell lines^25^. Based on this information, the overexpression and deletion of SVIL may be closely related to the occurrence of some tumors; however, research on SVIL is still very rare, especially in human tumor-related diseases. Therefore, it may be necessary to elucidate the role of eliminating this gene more urgently.

In the present study, we found that SVIL was differentially expressed in different tumor types. We studied SVIL expression in bladder cancer based on a combination of TCGA database, GEO database, bladder cancer cell lines (containing immortalized uroepithelial cells), and clinical specimens, which showed that SVIL expression was significantly downregulated at the mRNA level in both bladder cancer tissues compared to that in normal tissues. In contrast, in tumor tissues from patients with bladder cancer, elevated levels of SVIL expression were positively correlated with poorer clinicopathological features of bladder cancer. In addition, elevated SVIL expression levels in tissues of bladder cancer patients also had statistically significant correlation with poorer OS, DFS, and DSS in bladder cancer as well. Univariate and multivariate Cox regression analyses showed that SVIL level was an independent prognostic factor in patients with bladder cancer. In addition, the ROC analysis provided high confidence in the diagnostic value of SVIL in patients with bladder cancer.

Although SVIL did not show a linear decreasing trend in non-bladder cancer tissues, early bladder tissues, and advanced bladder cancer tissues, it still made a good prediction for differentiating between patients and non-patients, and between early and advanced patients. We speculate that this phenomenon is related to SVIL-encoded supervillin, an F-actin binding protein that may be involved in the development and formation of the bladder musculature and is important for the development of the maintained muscles^26^. In bladder carcinogenesis, the expression of this protein is significantly downregulated by certain mechanisms, leading to tumorigenesis. However, in advanced tumors, tumor aggressiveness and proliferative activity are significantly increased, which may prompt higher expression of supervillin in advanced tumor cells to maintain cell division and promote tumor cell invasion into the muscle layer. Anillin providing a synergistic role in this process^27^, which is also consistent with previous reports on the role of anillin in bladder cancer^28,29^. Therefore, although this nonlinear expression trend of SVIL may not be as clear and recognizable as other molecules with linear trends, we believe that SVIL still plays a role in predicting bladder cancer development and progression because of its multiple roles in SVIL muscle development and tumor progression.

To further explore the potential role of SVIL in bladder cancer, based on TCGA-BLCA dataset, we performed GO, KEGG analysis of SVIL co-expressed genes and GSEA analysis of SVIL. GO analysis revealed that the pathways such as skin and muscle development, adhesion molecules, receptor ligand activity, and oxidoreductase activity were significantly enriched, whereas the calcium signaling pathway, chemical carcinogen, and cytochrome P450 metabolic pathway were significantly enriched in KEGG analysis. The receptor ligand activity pathway is believed to be involved in the occurrence and development of many tumors such as renal cancer^30^, breast cancer^31^, and hepatocellular carcinoma^32^. In bladder cancer, receptor ligand activity is also widely involved in the occurrence and development of tumors and has become a promising target for the treatment of bladder cancer^33,34^. Chemical carcinogens are ubiquitous. Chemical carcinogens are involved in every biological stage of tumor cells, including initiation, promotion, and progression^35^. Bladder cancer is also closely related to a variety of chemical carcinogens such as cigarettes^36,37^ and polycyclic aromatic hydrocarbons^38^. Since SVIL and its co-expressed genes may be involved in receptor ligand activity and chemical carcinogen pathways and these pathways are closely related to the occurrence and development of tumors, especially bladder cancer, we can speculate that SVIL may regulate the occurrence and progression of bladder cancer through multiple pathways.

In GSEA analysis, several pathways corresponding to the SVIL overexpression phenotype were significantly enriched, including stem cells, multiple tumor progression characteristics, and bladder cancer pathway. It may be related to the protein encoded by SVIL, an F-actin-binding protein. Phosphorylated SVIL may act as a molecular link between the central spindle and contractile ring to coordinate the activation of myosin II^39^. The protein expressed by SVIL is important for the integrity of human muscle fibers, and its deletion leads to myopathy with myofibrillar disorganization^40^. Since the bladder is a muscular organ with strong systolic and diastolic functions, stable expression of SVIL may also be of great significance for the development of the bladder and the maintenance of normal functions. The absence of SVIL may promote or accelerate the occurrence of bladder cancer. In the low SVIL expression group, the rapamycin sensitivity, respiratory electron transport chain, oxidative phosphorylation pathways were significantly enriched, suggesting that cells in the low SVIL expression group may face more severe hypoxia and oxidative stress microenvironment. Hypoxia is a common feature of TME^41^. However, hypoxia can also promote angiogenesis in the microenvironment through rapamycin-dependent signal transduction^42^. Rapamycin plays a significant positive role in regulating hypoxia-induced stress in human cancer cells^43^. Fechner et al. reported that rapamycin can inhibit the growth of human bladder cancer cell lines in vitro and the release of vascular factors. Although it cannot induce apoptosis in bladder cancer cells, it causes cell cycle arrest^44^. This suggested that the low SVIL expression group may be more sensitive to rapamycin treatment.

We described the correlation between the level of immune cell infiltration and SVIL expression in bladder cancer. The results showed that SVIL was positively correlated with macrophage, eosinophil and mast cell infiltration and negatively correlated with NK CD56^bright^ cells. The immune cells in the TME and their related immune regulation mechanisms have a profound impact on the development of tumors, efficacy of cancer treatment, and a close relationship with clinical results^17,20^. This situation is similar for bladder cancer^45–47^. For example, the levels of neutrophil peptide-1, -2, and -3 produced by neutrophils increase in bladder cancer and may promote tumor angiogenesis and growth, while the main role of macrophages is mediated by proinflammatory cytokines IL-6 and TNF-α^17,20^. SVIL can regulate the movement of macrophages, and knockdown of SVIL can cause cell cycle arrest of macrophages. In addition, SVIL participates in the LPS-induced inflammatory response of IL-6, IL-1β, and TNF-α in macrophages^48^. Although M2 macrophages in bladder cancer can directly respond to the tumor-specific immune response in BCG-induced bladder cancer, they are the main members of the bladder cancer TME that inhibit tumor growth and metastasis^49^. However, M2 macrophage polarization is usually associated with the bladder cancer progression and poor prognosis^50^. Hanada et al.^51^ reported that bladder cancer patients with a high tumor-associated macrophage (TAM) count had a significantly higher rate of bladder resection, distant metastasis, vascular invasion and poor 5-year survival rate than the patients with low TAM count. Tumor-associated eosinophils are an unusual phenomenon. Although it was previously believed that eosinophils in tumor-associated tissues had a good prognosis^52^, Harsha et al.^53^ proposed a completely opposite view. They found that patients with diffuse eosinophil infiltration in tumor tissue pathology had rapid deterioration of their condition within a few days. In a study involving 156 patients with recurrent and non-recurrent bladder cancer, it was found that patients with higher eosinophil infiltration at the primary tumor site had a higher probability of tumor recurrence within 6 months^54^. SVIL expression also positively correlated with mast cell infiltration. It has been reported that a high degree of mast cell infiltration is an independent risk factor for the poor prognosis of MIBC^55^, which may be related to mast cells promoting the metastasis of bladder cancer^56^. It has also been reported that mast cells promote angiogenesis in the TME of bladder cancer and accelerate tumor progression and invasion through immunosuppression and epithelial-mesenchymal transition (EMT)^54^. These conclusions are consistent with our results. As the expression of SVIL in high-grade bladder cancer patients is higher than that in low-grade bladder cancer patients, it is related to the infiltration of macrophages, eosinophils, and mast cells at higher levels, resulting in a poor immune state. The expression level of SVIL is significantly negatively correlated with CD56^bright^ NK cells, which means that a higher level of SVIL expression may be accompanied by a lower level of CD56^bright^ NK cells infiltration, whereas NK cells can kill tumors through non-MHC restriction, especially CD56^bright^ NK cells. Compared to CD56^dim^ NK cells, CD56^bright^ NK cells in tumors show stronger cytotoxicity and are associated with better OS in bladder cancer patients^57^.

Finally, our study showed a correlation between SVIL and PD-1/PD-L1 related molecules. PD-1 is one of several key receptors that mediate immune escape. PD-L1 is a drug that targets the ligand of PD-1 and has been successfully applied to patients with metastatic bladder cancer, but more research is needed appraise the positive standard of PD-L1, explore its use as a biomarker, and expand its indication in bladder cancer^54^. In our study, the expression of SVIL was positively correlated with the expression of PDCD1, CD274, CTLA4, and LAG3, which suggests that although in bladder cancer patients, high levels of SVIL are associated with poor prognosis and immune infiltration status, they may have a better response to immune checkpoint treatment and can obtain better benefits from it. It is worth noting that our current research data provide evidence that SVIL is involved in regulating immune invasion in the local TME of bladder cancer. However, unbiased methods are needed to further evaluate the function and pathway of SVIL in the immune microenvironment of bladder cancer.

The importance and originality of our analysis in this study lies in providing the first systematic investigation of the relationship between SVIL and bladder cancer, which, to some extent, fills the gap in SVIL-related research in bladder cancer. However, this study still has some limitations. First, our research mainly relies on bioinformatics analysis, and although we have verified the mRNA expression in bladder cancer cell lines and patient tissues, and verified the difference of SVIL expression in bladder cancer tissues through tissue microarray with IHC. But the function and mechanism of SVIL in bladder cancer should be verified using a considerable number of in vivo and in vitro experiments. Secondly, the databases used in our research are limited; therefore, our results should be verified using multiple datasets. Finally, our study had the inherent limitations of a retrospective study design. Whether SVIL can be used as a diagnostic or prognostic marker that combines sensitivity and specificity or as a marker for immunotherapy, a large sample of prospective research is needed to confirm our findings.

## Conclusion

In conclusion, compared with the matched normal tissue adjacent to the cancer, SVIL in bladder cancer tissue was significantly reduced, but in the bladder cancer tissue group, high T stage and high-level bladder cancer tissue had higher SVIL (but still significantly lower than normal tissue). In bladder cancer, high expression of SVIL was closely related to the late case stage and poor prognosis, including OS, DSS, and DFS. ROC analysis revealed that SVIL can be used as a predictive marker to distinguish normal tissues from bladder cancer tissues. Univariate/multivariate Cox regression analysis revealed that an increase in SVIL levels was an independent adverse prognostic factor in bladder cancer. In addition, SVIL regulates and profoundly affects the occurrence and development of bladder cancer by participating in a variety of signaling pathways, including the stem cell pathway, receptor ligand activity, multiple cancer invasion characteristics, and other pathways, and by co-regulating immune cell invasion and the expression of PD-1 related molecules. However, further in vivo and in vitro experiments are required to verify our findings.

## Data Availability

The datasets presented in this study can be found in online repositories. The names of the repository/repositories and accession number(s) can be found in the article. The link of GSE13507 dataset is ‘https://www.ncbi.nlm.nih.gov/geo/query/acc.cgi?acc=GSE13507’ (Jun-13, 2023), while the access link of TCGA is ‘https://portal.gdc.cancer.gov/’ (Jun-13, 2023).

## References

[1] Siegel R, Miller K, Fuchs H, Jemal A (2022). Cancer statistics, 2022. CA Cancer J. Clin..

[2] Siegel R, Miller K, Fuchs H, Jemal A (2021). Cancer Statistics, 2021. CA Cancer J. Clin..

[3] Chen W (2016). Cancer statistics in China, 2015. CA Cancer J. Clin..

[4] Ritch C (2020). Use and validation of the AUA/SUO risk grouping for nonmuscle invasive bladder cancer in a contemporary cohort. J. Urol..

[5] Alfred Witjes J (2017). Updated 2016 EAU guidelines on muscle-invasive and metastatic bladder cancer. Eur. Urol..

[6] Neapolitan R, Horvath C, Jiang X (2015). Pan-cancer analysis of TCGA data reveals notable signaling pathways. BMC Cancer.

[7] Chen Y (2003). F-actin and myosin II binding domains in supervillin. J. Biol. Chem..

[8] Pestonjamasp K, Pope R, Wulfkuhle J, Luna E (1997). Supervillin (p205): A novel membrane-associated, F-actin-binding protein in the villin/gelsolin superfamily. J. Cell Biol..

[9] Chen X (2017). A novel splice variant of supervillin, SV5, promotes carcinoma cell proliferation and cell migration. Biochem. Biophys. Res. Commun..

[10] Chen X (2018). Supervillin promotes epithelial-mesenchymal transition and metastasis of hepatocellular carcinoma in hypoxia via activation of the RhoA/ROCK-ERK/p38 pathway. J. Experim. Clin. Cancer Res..

[11] Fang Z, Luna E (2013). Supervillin-mediated suppression of p53 protein enhances cell survival. J. Biol. Chem..

[12] Zhao C (2020). Supervillin promotes tumor angiogenesis in liver cancer. Oncol. Rep..

[13] Li T (2020). TIMER2.0 for analysis of tumor-infiltrating immune cells. Nucleic Acids Res..

[14] Kim W (2010). Predictive value of progression-related gene classifier in primary non-muscle invasive bladder cancer. Mol. Cancer.

[15] Lee J (2010). Expression signature of E2F1 and its associated genes predict superficial to invasive progression of bladder tumors. J. Clin. Oncol..

[16] Subramanian A (2005). Gene set enrichment analysis: A knowledge-based approach for interpreting genome-wide expression profiles. Proc. Natl. Acad. Sci. U. S. A..

[17] Bindea G (2013). Spatiotemporal dynamics of intratumoral immune cells reveal the immune landscape in human cancer. Immunity.

[18] Kanehisa M, Goto S (2000). KEGG: Kyoto encyclopedia of genes and genomes. Nucleic Acids Res..

[19] Kanehisa M, Sato Y, Kawashima M, Furumichi M, Tanabe M (2016). KEGG as a reference resource for gene and protein annotation. Nucleic Acids Res..

[20] Hanahan D, Coussens L (2012). Accessories to the crime: Functions of cells recruited to the tumor microenvironment. Cancer Cell.

[21] Martin-Doyle W, Kwiatkowski D (2015). Molecular biology of bladder cancer. Hematol./Oncol. Clin. N. Am..

[22] Guo L, Ding L, Tang J (2021). Identification of a competing endogenous RNA axis "SVIL-AS1/miR-103a/ICE1" associated with chemoresistance in lung adenocarcinoma by comprehensive bioinformatics analysis. Cancer Med..

[23] Mukherjee S, Sudandiradoss C (2021). Transcriptomic analysis of castration, chemo-resistant and metastatic prostate cancer elucidates complex genetic crosstalk leading to disease progression. Funct. Integr. Genomics.

[24] Lobl MB, Clarey D, Schmidt C, Wichman C, Wysong A (2021). Analysis of mutations in cutaneous squamous cell carcinoma reveals novel genes and mutations associated with patient-specific characteristics and metastasis: A systematic review. Arch. Dermatol. Res..

[25] Pope RK (1998). Cloning, Characterization, and Chromosomal Localization of Human Supervillin (SVIL). Genomics.

[26] Di L, Xin YQ, Wu X, Hu DX, Xiao QC (2018). Piperlongumine suppresses bladder cancer invasion via inhibiting epithelial mesenchymal transition and F-actin reorganization. Biochem. Biophys. Res. Commun..

[27] Smith TC (2013). Supervillin binding to myosin II and synergism with anillin are required for cytokinesis. Mol. Biol. Cell.

[28] Zeng S (2017). Transcriptome sequencing identifies ANLN as a promising prognostic biomarker in bladder urothelial carcinoma. Sci. Rep..

[29] Chen S, Gao Y, Chen F, Wang T (2022). ANLN serves as an oncogene in bladder urothelial carcinoma via activating JNK signaling pathway. Urologia Internationalis.

[30] Liu X, Wang J, Sun G (2015). Identification of key genes and pathways in renal cell carcinoma through expression profiling data. Kidney Blood Press. Res..

[31] Huan J (2014). Insights into significant pathways and gene interaction networks underlying breast cancer cell line MCF-7 treated with 17β-estradiol (E2). Gene.

[32] Liu Z, Gartenhaus R, Tan M, Jiang F, Jiao X (2008). Gene and pathway identification with Lp penalized Bayesian logistic regression. BMC Bioinform..

[33] Mansure J, Nassim R, Kassouf W (2009). Peroxisome proliferator-activated receptor gamma in bladder cancer: A promising therapeutic target. Cancer Biol. Ther..

[34] Nakashiro K (2001). Role of peroxisome proliferator-activated receptor gamma and its ligands in non-neoplastic and neoplastic human urothelial cells. Am. J. Pathol..

[35] Oliveira P (2007). Chemical carcinogenesis. Anais da Academia Brasileira de Ciencias.

[36] Hemminki K, Försti A, Hemminki A, Ljungberg B, Hemminki O (2021). Incidence trends in bladder and lung cancers between Denmark, Finland and Sweden may implicate oral tobacco (snuff/snus) as a possible risk factor. BMC Cancer.

[37] Anastasiou I (2010). Patient awareness of smoking as a risk factor for bladder cancer. Int. Urol. Nephrol..

[38] Clavel J, Mandereau L, Limasset J, Hémon D, Cordier S (1994). Occupational exposure to polycyclic aromatic hydrocarbons and the risk of bladder cancer: A French case-control study. Int. J. Epidemiol..

[39] Hasegawa H (2013). The role of PLK1-phosphorylated SVIL in myosin II activation and cytokinetic furrowing. J. Cell Sci..

[40] Hedberg-Oldfors C (2020). Loss of supervillin causes myopathy with myofibrillar disorganization and autophagic vacuoles. Brain J. Neurol..

[41] Mazumdar J, Dondeti V, Simon M (2009). Hypoxia-inducible factors in stem cells and cancer. J. Cell Mol. Med..

[42] Humar R, Kiefer F, Berns H, Resink T, Battegay E (2002). Hypoxia enhances vascular cell proliferation and angiogenesis in vitro via rapamycin (mTOR)-dependent signaling. FASEB J..

[43] Abraham R (2004). mTOR as a positive regulator of tumor cell responses to hypoxia. Curr. Top. Microbiol. Immunol..

[44] Fechner G, Classen K, Schmidt D, Hauser S, Müller S (2009). Rapamycin inhibits in vitro growth and release of angiogenetic factors in human bladder cancer. Urology.

[45] Thompson D, Siref L, Feloney M, Hauke R, Agrawal D (2015). Immunological basis in the pathogenesis and treatment of bladder cancer. Expert Rev. Clin. Immunol..

[46] Li F, Guo H, Wang Y, Liu B, Zhou H (2020). Profiles of tumor-infiltrating immune cells and prognostic genes associated with the microenvironment of bladder cancer. Int. Immunopharmacol..

[47] Hatogai K, Sweis R (2020). The tumor microenvironment of bladder cancer. Adv. Exp. Med. Biol..

[48] Zhou J (2022). Supervillin contributes to LPS-induced inflammatory response in THP-1 Cell-derived macrophages. Inflammation.

[49] Sharifi L (2019). A review on the role of M2 macrophages in bladder cancer; pathophysiology and targeting. Int. Immunopharmacol..

[50] Martínez V (2017). BMP4 induces M2 macrophage polarization and favors tumor progression in bladder cancer. Clin. Cancer Res..

[51] Hanada T (2000). Prognostic value of tumor-associated macrophage count in human bladder cancer. Int. J. Urol..

[52] Lowe D, Jorizzo J, Hutt MS (1981). Tumour-associated eosinophilia: A review. J. Clin. Pathol..

[53] Hanada T, Nakagawa M, Emoto A, Nomura T, Nomura Y (2010). Prognostic value of tumor-associated macrophage count in human bladder cancer. Int. J. Urol..

[54] Perlin DS (2017). A review of the PD-1/PD-L1 checkpoint in bladder cancer: From mediator of immune escape to target for treatment. Urol. Oncol..

[55] Zhu Y, Liu Z, Fu H, Zhang J, Ye D (2018). Tumor stroma-infiltrating mast cells predict prognosis and adjuvant chemotherapeutic benefits in patients with muscle invasive bladder cancer. Eur. Urol. Suppl..

[56] Qun (2016). Recruited mast cells in the tumor microenvironment enhance bladder cancer metastasis via modulation of ERβ/CCL2/CCR2 EMT/MMP9 signals. Oncotarget.

[57] Mukherjee N, Ji N, Hurez V, Curiel TJ, Montgomery MO, Braun AJ, Nicolas M, Aguilera M, Kaushik D, Liu Q, Ruan J (2018). Intratumoral CD56bright natural killer cells are associated with improved survival in bladder cancer. Oncotarget.

