# Author Correction: Duality of the SVIL expression in bladder cancer and its correlation with immune infiltration

**DOI:** 10.1038/s41598-024-52867-x

**Published:** 2024-01-29

**Authors:** Zhenyu Nie, Na Guo, Yanling Peng, Yuanhui Gao, Hui Cao, Shufang Zhang

**Affiliations:** Central Laboratory, Affiliated Haikou Hospital of Xiangya Medical College, Central South University, Haikou, 570208 Hainan China

Correction to: *Scientific Reports* 10.1038/s41598-023-41759-1, published online 05 September 2023

The original version of this Article contained an error in the Funding section.

“This work was supported by the Finance Science and Technology Project of Hainan Province (grant numbers ZDYF2021SHFZ096).the National Nature Science Foundation of China (grant numbers 82160531).”

now reads:

“This work was supported by the Finance Science and Technology Project of Hainan Province (grant numbers ZDYF2021SHFZ249).the National Nature Science Foundation of China (grant numbers 82160531).”

The original Article has been corrected.

